# Effects of cigarette smoking on semen quality, reproductive hormone levels, metabolic profile, zinc and sperm DNA fragmentation in men: results from a population-based study

**DOI:** 10.3389/fendo.2023.1255304

**Published:** 2023-10-18

**Authors:** Ludmila Osadchuk, Maxim Kleshchev, Alexander Osadchuk

**Affiliations:** Department of Human Molecular Genetics, Federal Research Center ‘Institute of Cytology and Genetics’, the Siberian Branch of the Russian Academy of Sciences, Novosibirsk, Russia

**Keywords:** cigarette smoking, semen quality, sperm DNA fragmentation, reproductive hormones, metabolic profile, seminal zinc

## Abstract

**Background:**

Cigarette smoking seems to have a negative impact on men’s reproductive health, but our knowledge of its effects on the reproductive function of Russian men is still very limited. The purpose of this study was to evaluate the effect of cigarette smoking on semen quality, including sperm DNA fragmentation, hormonal, zinc and metabolic status in young men from the general multi-ethnic Russian population (n=1,222, median age 23 years) and to find out the ethno-specific effects of smoking by comparing male groups of different ethnicity.

**Methods:**

Each participant filled out a standardized questionnaire, provided one blood and semen sample. Semen parameters, serum reproductive hormones, lipids, glucose, uric acid and seminal zinc were analyzed. Participants were classified as smokers (n=450) and non-smokers (n=772), and smokers were stratified into moderate (≤10 cigarettes/day) and heavy (>10 cigarettes/day) smokers.

**Results:**

In the entire study population, heavy smokers were characterized by a decrease in semen volume, total sperm count, sperm concentration and motility, and an increase in sperm DNA fragmentation and teratozoospermia compared with non-smokers (p<0.05). There was also a reduction in the serum and seminal zinc level as well as an impairment in metabolic health in smokers compared with non-smokers (p<0.05). No significant differences between smokers and non-smokers were found for serum levels of LH, FSH, inhibin B, testosterone and estradiol. In the second part of our study, the most numerous ethnic groups of Slavs (n=654), Buryats (n=191), and Yakuts (n=125) were selected from the entire study population. Among three ethnic groups, the smoking intensity was higher in Slavs than in Buryats or Yakuts suggesting a greater tobacco addiction in Slavs than in Asians. A decrease in semen parameters and seminal zinc levels, and an increase in sperm DNA fragmentation and teratozoospermia was observed only in smoking Slavs (p<0.05); moderate decrease in testosterone and increase in triglyceride levels were revealed in smoking Yakuts (p<0.05), but no significant changes were detected in smoking Buryats.

**Conclusion:**

We concluded that cigarette smoking has an ethno-specific effect on male reproductive function, probably due to the different activity of the seminal antioxidant system, which is yet to be elucidated.

## Introduction

In recent decades, the demographic crisis observed in highly developed countries, including Russia, has been combined with a progressive decline in the reproductive potential of human populations, which raises the question of the underlying causes of this phenomenon. In different regions of the world, adverse secular trends in male reproductive health has been observed, which are expressed in poor semen quality, an increase in the proportion of male factor in infertile couples and the growth of congenital anomalies of the male reproductive system, resulting to infertility (1–4). In addition to a reduction of semen volume, sperm concentration and motility and an increase in sperm morphological abnormalities, several epidemiological studies have indicated a population-level decline in testosterone, suggesting that all these facts may be interrelated (5–7).

Since adverse changes in male fertility have occurred in a short period of time and in parallel in different parts of the world, the decline in semen quality is probably due to environmental rather than genetic factors (4). In recent years, it has been well documented that adverse changes in male fertility seem to be primarily associated with primarily anthropogenic environmental pollution, occupational exposure and individual lifestyle (8–11). Lifestyle, especially bad habits such as smoking, alcohol consumption, physical inactivity or excessive nutrition, have a negative impact not only on general health, but also reduce sperm quality and can lead to male sub-fertility or infertility (11–13). Some authors believe that the worldwide decline in men’s reproductive potential might be due, at least in part, to lifestyle factors (4, 8, 9).

Tobacco smoking is considered among the most significant lifestyle factors affecting male reproductive function. Smoking is still widespread all over the world, including Russia. According to the World Health Organization, 22.3% of the global population, 36.7% of men and 7.8% of women, used tobacco in 2020 (14). Its prevalence in the United States is about 21-22% among men of reproductive age (9, 15). The Russian Public Opinion Research Center (VCIOM) reports that the proportion of smokers in Russia has remained unchanged over the past five years and today it is a third of total number of citizens (33%), while smoking men are much more than women (47% vs 21%) (16).

It is generally recognized that tobacco smoking has a negative impact on overall health and is one of the leading preventable causes of death. Tobacco smoking is associated with more than 27 diseases, including respiratory and cardiovascular diseases, lung, kidney, urinary bladder, pancreas cancer, type 2 diabetes and other medical conditions (17, 18). Despite the fact that smoking is a well-established cause of many hard diseases, the mechanism by which smoking causes such a wide range of diseases is not fully elucidated. Although many are aware of the dangers of tobacco use, smoking continues to be a public health concern. Only recently, due to the growth of male infertility and subfertility, increased attention has been paid to the effects of smoking on men’s reproductive health (19). Cigarette smoking is a potential risk factor for impaired male fertility and the relationship between smoking and male infertility is actively being discussed. A decrease in semen volume, sperm concentration, viability, motility and normal morphology has been shown in heavy tobacco smokers (8, 9, 19–22). Moreover, in ART programs, chronic smoking of male partner was related to poor embryo quality and the reduced success of *in vitro* fertilization (IVF) and intracytoplasmic sperm injection (ICSI) (8, 23–25). Although many studies have reported a decrease in one or more conventional semen parameters or altered sperm functions in tobacco smokers, some studies have not established or shown very weak negative changes in semen quality, especially in infertile and sub-fertile men (20, 21, 23, 26, 27).

Based on studies that evaluated semen parameters in accordance with the WHO guidelines (28), it can be concluded that the effects of tobacco smoking lead to a more pronounced poor semen quality in healthy men or men from the general population (19, 29–32) than in infertile men or men from infertile couples (33–37). Moreover, it has been shown that tobacco smoking can affect the conventional semen parameters in a dose-dependent way, since the impairment of semen quality was more pronounced in heavy smokers then in moderate or mild smokers, which indicates more severe reproductive consequences with longer or intensive smoking (19, 29, 30, 38, 39).

More than 7,000 chemicals and toxins have been identified in tobacco smoke including polycyclic aromatic hydrocarbons, of which many are proven carcinogens and mutagens (8, 20, 22, 40, 41). The main pharmacological hazardous substances of tobacco – the alkaloids nicotine and its metabolite cotinine - can penetrate the blood-testis barrier and subsequently affect the process of spermatogenesis, reducing sperm concentration, total sperm count, and sperm motility (19–23). Smoking has been shown to increase genetic and epigenetic aberrations in spermatozoa, including oxidative DNA damage, chromatin packing abnormalities, chromosomal alterations, mutations, polymorphisms, epigenetic alterations such as DNA methylation and dysregulation of mRNA expression, and all of that can influence sperm functions and men’s fertility (8, 20, 22, 37, 41–46). Recent research data suggest that genetic and epigenetic changes in spermatozoa that are caused by the genotoxic components of tobacco can be transmitted to offspring (19, 20, 22, 40–43).

Chemical compounds with which smoking might affect the sperm DNA integrity include cigarette smoke components such as cadmium, lead, arsenic, carbon monoxide, nicotine and cotinine (a metabolite of nicotine), which are directly absorbed into the systemic circulation and accumulated in seminal plasma, initiate oxidative stress and dramatically affect seminal parameters, including sperm DNA fragmentation (22, 40–43). Oxidative damage is the most common cause of sperm DNA fragmentation, which can impair sperm functions, leading to a decrease in male fertility (20). Some authors have suggested using the degree of DNA integrity as an additional parameter of semen quality and a prognostic factor of male fertility (42). In 2021, the WHO laboratory guidelines (47) also recommended the analysis of sperm DNA fragmentation as a new functional test for an extended study of sperm quality. In addition to tobacco smoking, other lifestyles are associated with increased sperm DNA fragmentation, including excessive alcohol consumption, obesity, advanced age, urological diseases, type 2 diabetes (42).

The effect of tobacco smoking on semen parameters can be mediated not only by direct toxic effects, but also by changes in hormonal secretion and metabolism (20, 22, 48, 49). Tobacco smoke can induce a dysregulation of the endocrine system, which leads to problems with male fertility. To date, research assessing the correlation between cigarette smoke exposure and reproductive hormones has shown inconsistent results. Some studies revealed significantly higher levels of testosterone and/or free testosterone in smokers compared to non-smokers with a dose-response pattern (13, 32, 38, 49–52). On the contrary, others reported a decrease in testosterone concentrations in tobacco smokers (29, 53) or did not find any differences in the hormonal level between smokers and non-smokers (54, 55). Although many studies have found increased testosterone levels among smokers, the mechanism by which smoking increases testosterone levels remains unclear. The increase in testosterone levels in smokers may be due to a change in the ability of SHBG to bind testosterone (56, 57). Zhao et al. (49) suggested that cotinine (a metabolite of nicotine) can inhibit the testosterone degradation, which leads to the testosterone accumulation in smokers. Moreover, higher total testosterone levels in smokers than in non-smokers were positively associated with number of cigarettes smoked, but with age the positive relationship between smoking and testosterone weakened (58). Interestingly, higher testosterone concentrations in smokers were associated with higher sexual activity, although long-term experience of smoking has a higher risk of erectile dysfunction (20, 59, 60).

Few studies have investigated the relationship between tobacco smoking and pituitary hormones. Differences in FSH and LH levels between non-smokers and smokers were very often insignificant suggesting that there is no effect of tobacco smoking on the hypothalamic-pituitary axis (13, 32, 38, 61). Meanwhile, Blanco-Muñoz with colleagues showed that active smokers had higher LH and testosterone levels, although they were unable to detect any changes in estradiol, inhibin B and FSH levels (62). One recent study found that FSH levels were higher in smokers than in non-smokers, while testosterone levels were lower and LH levels were no different (53). Furthermore, Mitra with colleagues found that serum FSH and LH levels were higher in smokers compared to non-smokers, while testosterone levels decreased significantly with increased smoking (63).

Several studies have documented the damaging effects of tobacco smoking on the antioxidant defense system due to increased oxidative stress (20, 22, 40, 41, 64). Cigarette smoke contains high concentrations of nitric oxide, peroxynitrite, free radicals, and can potentially induce the production of reactive oxygen species (ROS) in the human body. Oxidative stress occurs when the antioxidant system can no longer counteract the ROS formation. Excessive generation of ROS damages the structural and functional parameters of sperm, including polyunsaturated fatty acids, proteins, nucleic acids, which are potential targets of ROS. Antioxidants, including superoxide dismutase, glutathione peroxidase, catalase, play an important defensive role in neutralizing ROS. The trace element zinc is an essential element as more than 300 enzymes require zinc for their function, and plays an important role in functioning the male reproductive system, including sperm physiology and the antioxidant defense mechanism (65–67). Several studies have reported that seminal zinc levels were closely associated with semen parameters in men, so zinc deficiency may be an important risk factor for lowered semen quality (65–69). Zinc has antioxidant properties that counteract the excessive production of ROS, in particular, it is a necessary component of superoxide dismutase, the most active antioxidant enzyme in the semen. Zinc depletion or deficiency (particularly the actual levels of seminal zinc) is proved to be a serious side effect of cigarette smoking (69, 70). Taha with colleagues observed that fertile non-smokers demonstrated higher progressive sperm motility, seminal zinc level, lower levels of sperm DNA fragmentation and seminal ROS compared to fertile smokers (64). Similarly, in infertile couples, it was found that the seminal zinc concentration and semen parameters (sperm concentration, motility and morphology) were lower in smokers than in non-smokers, indicating that seminal zinc may be involved in the mediating effects of tobacco smoking on semen parameters (70). In a large cohort of men from infertile couples, it was observed that sperm progressive motility, abnormal head rates, sperm viability, seminal zinc concentration were significantly reduced, as well as the DNA fragmentation rate was markedly increased in heavy smokers compared with non-smokers (35). When comparing groups of male volunteers, the smoking group compared with non-smokers showed a lower sperm concentration, the proportion of actively motile and morphologically normal sperm. Seminal zinc levels were also reduced in smokers compared to non-smokers, although there was no significant difference in serum zinc levels between smokers and non-smokers (69). The above studies clearly show the negative impact of tobacco smoking on the seminal zinc level, which may contribute to the pathogenesis of the adverse effects of smoking on semen parameters.

Tobacco smoking appears to negatively affect lipid, glucose and protein metabolism. Studies have shown an adverse effect of tobacco smoking on the lipid profile (lower HDL, higher LDL and triglycerides), while advanced age and higher BMI significantly enhance this relationship (71). Nicotine suppresses appetite and can reduce food intake and body weight, so smokers tend to be thinner and have a lower BMI than non-smokers, at that the lipolytic effect of smoking is attributed to the nicotine component through the release of catecholamines (71). Smoking can be associated with impaired in insulin action and glucose regulation, increasing risk for type 2 diabetes (71). Some men smoke because they consider their habit as a method of weight control, however, there is evidence that a part of heavy smokers has a higher BMI and a larger waist circumference than non-smokers, while the combination of smoking with abdominal fat accumulation increases the risk of metabolic syndrome, type 2 diabetes and cardiovascular diseases (72).

Most of the current researches concerning the effect of tobacco smoking on male reproductive function were carried out on specially selected small groups, in most cases with a history of infertility and/or long-term smoking experience, and very few studies have examined the effect of tobacco smoking in population-based cohorts. At the same time, a larger sample size increases its external validity and allows to control numerous potential confounding variables. Moreover, a large-scale population study can clarify previously unresolved issues, in particular, to identify new relationships due to ethnic origin and traditions. Although the exact associations tobacco smoking with the male fertility parameters are still insufficiently studied, our knowledge about its reproductive effects in Russian residents is even more limited, especially in men of active reproductive age. Thus, the purpose of this study was 1) to investigate whether tobacco smoking is associated with the semen quality parameters (total sperm count, concentration, motility, morphology, DNA integrity), the reproductive hormone and zinc levels, metabolic status in Russian men of reproductive age from the general multi-ethnic population and 2) to find out the ethno-specific effects of smoking when comparing different ethnic groups.

## Materials and methods

### Subjects

For the present study, 1,371 men were recruited from five Russian cities: Archangelsk, Novosibirsk, Kemerovo, Ulan-Ude, Yakutsk. The city of Archangelsk is located in European North of Russia within the circumpolar area, the cities of Novosibirsk and Kemerovo in Western Siberia; all three cities have a predominantly Slavic population (approximately 90-95%). The cities of Ulan-Ude and Yakutsk are located in Eastern Siberia. Buryats make up 32% of the total population of Ulan-Ude, and Yakut - 43% of the total population of Yakutsk. In all cities, the study design and standardized recruitment protocol were the same, which were described earlier (68, 73, 74). The study included volunteers from the general population, regardless of their fertility, most of the participants were undergraduate or postgraduate students, and university staff. Any man over the age of 17 who wanted to know his reproductive health and endocrine status was invited to participate in the study. All the participants were born or lived for at least 3-5 years in the cities prior to the study. Participants were recruited without restrictions on body weight and BMI. All the participants were informed about the study in various ways, including advertising on the Internet, on television or at special lectures on men’s health, with detailed information about the purpose and objectives of the study. Each participant filled in a standardized questionnaire with information on age, place of born, self-identified nationality, family status, some life style characteristics, including smoking consumption. All study subjects were voluntaries and did not received any financial compensation. Since our study population was multi-ethnic and consisted of men of different nationalities or descendants from ethnic mixed marriages, the questionnaire also included questions about the ethnicity of the participant’s parents and grandparents. To investigate ethnic differences in the effects of cigarette smoking, three most numerous ethnic groups were selected from our multi-ethnic study population. They were Slavs (n=654), Buryats (n=191) and Yakuts (n=125). Participants were stratified into the ethnic groups, taking into account self-identified ethnicity and ethnicities of their parents and grandparents. The participant was eligible if the ethnicity of himself and his relatives was the same. The data of participants were stored anonymously.

All participants were examined by an andrologist, and some participants were diagnosed with current urogenital disorders, which included clinical varicocele grade II, prostatitis, testicular cysts, hydrocele, and hypospadias. Body weight (kg), height, waist and hip circumference (cm) were measured, body mass index (BMI, kg/m^2^) was calculated. Testicular volume (ml) was estimated by a Prader orchidometer and was presented as bitesticular or paired testicular volume (BTV). Age calculated as the difference between year of attendance in study and self-reported year of birth. Each participant was asked about necessity of sexual abstinence for 2-7 days before the examination. Each participant provided both blood and semen sample. The fasting blood sample was drawn in the morning between eight and eleven a.m. before the semen sample was collected. Serum samples were stored at -40°С until an analysis for hormones, metabolites and zinc. Semen samples were collected by masturbation into disposable sterile plastic containers. The participants with the following characteristics as azoospermia, varicocele of II or III grade, orchitis, criptorchidism or the consequences of surgery for cryptorchidism, testis trauma, using drugs, chronic diseases in an acute phase were excluded from the study. Participants, who took anabolic steroids (reported themselves or indicated from the profile of reproductive hormones) or did not provide information about smoking status, as well as men with AZF microdeletions of the Y chromosome (known genetic causes of infertility) were also excluded. Totally, 1,222 participants (89.1% of recruited men) were included in the study. Participants were classified as smokers if they indicated that they currently smoke daily and reported an approximate number of cigarettes smoked per day, while non-smokers indicated that they had never smoked. Current smokers were further stratified into two groups, depending on the amount of cigarettes smoked daily: moderate (≤ 10 cigarettes/day) and heavy (> 10 cigarettes/day).

### Semen analysis

Sperm concentration (million/ml), semen volume (ml), total sperm count (million/ejaculate), a proportion of morphologically normal (%) and progressively motile sperm (%), the teratozoospermia index (TZI) were analyzed according to the WHO laboratory manual for the examination and processing of human semen (28). Semen analysis was described earlier (68, 73, 74). The semen samples were kept at 37^0^C for 1 hr. for liquefaction. Ejaculate volume was estimated by weighing the collection container and subtracting the weight of the empty preweighed container, assuming that 1 ml of ejaculate weighs 1 g. To determine the sperm concentration, 100 μl of well-mixed ejaculate was diluted in 400 μl of a solution (5% NaHCO3; 0.35% formaldehyde; 0.025% trypan blue in distilled water). Staining was carried out for 1 hr. at room temperature, after which the samples were stored in the refrigerator at a temperature of +40°C for subsequent counting. Sperm concentration was assessed using the Goryaev’s hemocytometer under light microscope (magnification ×400). Total sperm count was calculated by multiplying the individual’s sperm concentration by the semen volume.

A proportion of progressive motility sperm was estimated in native ejaculate using an automatic sperm analyzer SFA-500 (Biola, Russia). The sperm motility measurements were carried out three times for each sample and mean value was calculated. To assess sperm morphology, ejaculate smears were prepared, fixed by methanol and stained by using commercially available kits Diff-Quick (Abris plus, Russia) according to the manufacturer manual. Two hundred spermatozoa in each sample were examined for morphology with an optical microscope (Axio Skop.A1, Carl Zeiss, Germany) at ×1000 magnification with oil-immersion and the sperm morphological anomalies were listed according to the WHO laboratory manual (28). To determine the TZI, the total number of morphological defects determined was divided by the number of morphological abnormal sperm. Sperm morphology evaluations were done in duplicates in random and blind order and we reported here the percentage of sperm assessed as morphologically normal (%).

### Serum reproductive hormones and metabolites assay

Serum hormone concentrations were determined by enzyme immunoassay using commercially available kits Steroid IFA-Testosterone-01, Gonadotropin IFA-LH, Gonadotropin IFA-FSH (Alkor Bio, Russia), Estradiol-IFA (Xema, Russia) and Inhibin B Gen II ELISA (Beckman Coulter, USA). The ranges of evaluated concentrations for total testosterone (T), estradiol (E_2_), follicle-stimulating hormone (FSH), luteinizing hormone (LH), and inhibin B (InhB) were 0.2-50 nmol/L, 0.1-20 nmol/L, 2.0-100 mIU/mL, 20-90 mIU/mL, and 12-105 pg/ml respectively. The sensitivities for T, E_2_, FSH, LH, InhB were 0.2 nmol/ml, 0.025 nmol/L, 0.25 mIU/mL, 0.25 mIU/mL, 2.6 pg/ml, respectively. The intra- and interassay coefficients of variation were as follows: for T < 8.0%; E_2_ < 8.0%; FSH < 8.0%; LH < 8.0%, InhB ≤ 6.8%.

Serum concentrations of triglycerides (TG), total cholesterol (TC), high-density lipoprotein cholesterol (HDL-C), glucose and uric acid were determined by enzymatic colorimetric method using commercially available kits (Vector Best, Russia). According to this test system, the normal values of serum metabolite concentrations in adults were TG up to 1.70 mmol/l; TC up to 5.20 mmol/l; HDL-C for men in the range of 0.90-1.80 mmol/l; glucose 4.0-6.1 mmol/l; uric acid for men 200-420 µmol/l. Serum concentrations of low-density lipoprotein cholesterol (LDL-C) was calculated using the Friedewald formula (LDL-C = TC - HDL-C - TG/2.2).

### Serum and seminal plasma zinc assay

Seminal plasma was obtained by centrifugation of the cellular elements and was stored at -40°C. Serum and seminal zinc was determined by spectrophotometry and direct colorimetry without deproteinization using commercially available kits (Vital Development Corporation, Russia) as previously described (68). The seminal plasma diluted with 1:100 of deionized water before the analyses, the serum samples used without dilution. The total seminal zinc content was calculated by multiplying the individual’s seminal zinc concentration by the semen volume. The detection limit for zinc concentration was one μmol/l; the lineal range of zinc concentrations was 6.0 - 61.2 μmol/l; coefficient of variation was ≤ 5%. According to this test system, the normal values of serum zinc concentrations were 7.0-23.0 μmol/l in adults. Lower reference limit for seminal zinc content is 2.4 μmol/ejaculate (28).

All hormones, metabolites and zinc were assayed using photometer Multiskan Ascent (Thermo Electron Corporation, Finland). Spectral range 340-850 nm. Operating wavelengths for TG, glucose, uric acid and zinc – 540 nm; for TC, HDL-C – 492 nm; for all hormones – 450 nm.

### Sperm DNA fragmentation assay

The sperm DNA fragmentation was assessed using the Sperm Chromatin Structure Assay (SCSA) using flow cytometry as previously described (75). Immediately after ejaculate collection, semen aliquot (300 µL) was frozen and stores at -40°C until analysis. To analyze sperm DNA fragmentation, the sample diluted in TNE-buffer (0.01MTris, 1 mM EDTA, 0.15 MNaCl, pH 7.4) to the sperm concentration one million/ml and then 200 μl of acid buffer (0.1% Triton-X-100, 0.15M NaCl, 0.08 N HCl, pH 1.2) was added to 100 µl diluted ejaculate. After incubation for 30 s, 600 μl of dye solution containing 6 mg/L acridine orange, 0.2 M Na_2_HPO_4_, 1 mM EDTA (disodium), 0.15 M NaCl, 0.1 M citric acid (pH 6.0) was added. Sperm with red and green fluorescence were counted by fluorescence cytometer Guava Easy CyteMini (Guava, USA). Each sample was evaluated thrice (5000 cells in each estimation) and the mean value was used. Extent of DNA damage was expressed as the DNA fragmentation index (DFI%), which is the ratio of red (sperm with fragmented DNA) to total fluorescence. DFI% <15% is a normal percentage of sperm without chromatin damage, 15%≤ DFI% <27% is considerable chromatin damage and DFI% ≥27% is severe chromatin damage (76). The DNA fragmentation index was evaluated in randomly selected subjects from our entire study population (n=247, 20.2%), of which 200 participants were Slavs (145 non-smokers and 55 smokers).

### Statistical analysis

Statistical analysis of the data obtained was performed using the statistical package “STATISTICA” (version 8.0). The Kolmogorov-Smirnov test for normality was used. In the entire study population, all the studied parameters were not normally distributed. Descriptive statistics in the tables and in the text is presented as mean (SD) as well as median with 5th and 95th percentiles. Kruskal-Wallis ANOVA test was used to identify differences in age and anthropometric parameters between groups with different smoking status in the entire study population or between non-smokers and smokers in each ethnic group. Analysis of covariance (ANCOVA) was used to identify differences in semen, hormonal, metabolic and zinc parameters between groups with different smoking status or between groups of smokers and non-smokers in each ethnic group. The semen parameters, including DFI% and seminal zinc, were adjusted for the period of sexual abstinence, while serum hormonal, metabolic and zinc parameters were adjusted for age and BMI. Duncan’s test was used to determine statistical significance of the differences between the groups. A p value < 0.05 was regarded as statistically significant.

## Results

### General characteristics of the entire study population

Of the 1,222 participants, 654 (53.5%) men were Slavs (Russians, Belarusians, Ukrainians); 191 (15.6%) - Buryats; 125 (10.2%) - Yakuts; 25 (2.1%) were other ethnic origin; 227 (18.6%) were descendants from ethnic mixed marriages. The median age of men was 23 years, and the vast majority of participants (81.5%) were of reproductive age (18-30 years). Only 37.7% of the participants in our study population were married, and 16.5% of them had at least one or two children. Demographic and anthropometric parameters of the study subjects are summarized in **Table 1**. The entire study population was characterized by a normal BMI (median 23.4 kg/m^2^), although a part of the study population was overweight (25.3%) or obese (8.6%). More than one third of the men (36.8%) were cigarette smokers on daily basis (**Table 1**). According to the WHO laboratory manual (28), the median semen parameters of the participants were within the values accepted for normozoospermia, including the sperm concentration, motility and morphology (**Table 1**). The median sperm concentration was 52.49 million/ml; the lowered semen quality was detected in 39.2% of participants in accordance with the WHO, i.e. with sperm concentration less than 15 million/ml, progressive sperm motility less than 32% and normal morphology less than 4%. The median levels of LH, FSH, testosterone and metabolic parameters in our participants corresponded to normal reference ranges for men from European countries and the USA (77–79). The median concentrations of serum and seminal zinc were within the generally accepted normal values for Russian men (80). The median content of seminal zinc was 4.20 μmol/ejaculate, which is almost twice the WHO recommended lower reference value of 2.4 μmol/ejaculate (28).

**Table 1:** Anthropometric, semen, hormonal, and metabolic parameters of participants with different smoking status.

Parameters	Smoking status			Total (n=1222)
	Non-smokers (n=772)	Moderate smokers cigarettes/day ≤10 (n=274)	Heavy smokers cigarettes/day >10 (n=176)	
Age, years	24.9(7.0) **^a^** 23.0(18.0-39.0)	24.4(6.4) **^a^** 22.0(18.0-37.0)	27.7(8.0) **^b^** 26.0(18.0-43.0)	*25.2 (7.1)* *23.0 (18.0-39.0)*
Body weight, kg	75.4(13.6) **^a^** 73.6(57.2-102.0)	74.2(14.3) **^a^** 72.0(55.0-101.0)	78.5(14.9) **^b^** 76.3(59.5-106.0)	*75.5(14.0)* *74.0(56.5-102.0)*
Height, cm	177.6(7.2) **^a^** 177.5(166.0-189.0)	176.4(7.0) **^a^** 176.5(165.5-188.0)	176.6(6.7) **^a^** 176.0(165.0-187.5)	*177.2(7.1)* *177.0(166.0-188.5)*
Waist circumference, cm	83.5(10.2) **^a^** 82.0(70.0-103.0)	84.1(11.7) **^a^** 82.0(69.0-105.0)	87.9(12.4) **^b^** 87.0(70.0-107.0)	*84.2(11.0)* *82.0(70.0-105.0)*
Hip circumference, cm	97.9(7.9) **^a^** 97.0(87.0-112.0)	97.2(8.4) **^a^** 96.0(85.0-111.0)	99.2(8.5) **^b^** 98.0(87.0-114.0)	*98.0(8.1)* *97.0(86.0-112.0)*
BMI, kg/m^2^	23.8(3.8) **^a^** 23.3(18.8-31.2)	23.8(4.3) **^a^** 23.0(18.3-31.8)	25.2(4.6) **^b^** 24.5(19.1-32.0)	*24.0(4.0)* *23.4(18.7-31.4)*
BTV, ml	39.7(8.6) **^a^** 40.0(27.0-54.0)	38.8(7.4) **^a^** 40.0(28.0-50.0)	40.7(9.2) **^a^** 40.0(29.0-56.0)	*39.7(8.4)* *40.0(28.0-54.0)*
Smoking, cigarettes/day	0	6.6(3.0) **^a^** 7.0(1.3-10.0)	17.5(4.7) **^b^** 17.5(12.0-25.0)	*11.0(6.5)* *10.0(2.0-20.0)*
Semen volume, ml	3.6(1.6) **^a^** 3.4(1.5-6.5)	3.3(1.6) **^b^** 3.0(1.2-5.9)	3.3(1.6) **^b^** 3.0(1.1-6.3)	*3.5(1.6)* *3.3(1.3-6.4)*
Total sperm count, ×10^6/^ejaculate	196.8(188.4) **^a^** 156.8(26.1-450.5)	157.6(145.6) **^b^** 125.5(11.1-455.6)	147.2(118.3) **^b^** 112.7(9.9-410.2)	*176.1(171.5)* *136.4(15.0-444.5)*
Sperm concentration, ×10^6^/mL	55.15(40.74) **^a^** 44.31(10.11-136.93)	48.26(36.31) **^b^** 41.28(5.38-137.25)	47.36(35.25) **^b^** 39.75(4.63-117.85)	*52.49(39.16)* *43.00(7.44-131.58)*
Progressive motility, %	46.6(25.9) **^a^** 44.7(5.9-89.8)	43.6(26.7) **^ab^** 42.8(2.8-88.1)	39.3(26.0) **^b^** 36.1(3.2-83.6)	*43.8(26.6)* *41.7(3.3-89.0)*
Normal morphology, %	6.9(3.0) **^a^** 6.8(2.3-12.0)	6.4(3.0) **^b^** 6.1(1.9-11.5)	6.6(3.0) **^ab^** 6.8(2.0-11.3)	*6.6(3.0)* *6.5(2.0-11.8)*
TZI	1.48(0.12) **^a^** 1.47(1.32-1.69)	1.49(0.11) **^ab^** 1.48(1.32-1.71)	1.50(0.12) **^b^** 1.49(1.33-1.73)	*1.49(0.12)* *1.48(1.32-1.71)*
DFI, %	8.25(5.37) **^a^** 6.88(2.74-20.35) (n=180)	10.11(7.95) **^a^** 7.30(2.72-26.25) (n=36)	14.66(12.72) **^b^** 8.53(2.54-36.89) (n=29)	*9.28(7.29)* *7.06(2.70-25.74) (n=245)*
LH, mIU/mL	3.48(1.39) **^a^** 3.23(1.59-6.02)	3.68(1.53) **^a^** 3.38(1.48-6.33)	3.86(1.56) **^a^** 3.66(1.42-6.64)	*3.58(1.46)* *3.30(1.55-6.24)*
FSH, mIU/mL	3.91(2.20) **^a^** 3.55(1.50-7.86)	4.08(2.13) **^a^** 3.63(1.47-8.21)	4.16(2.78) **^a^** 3.55(1.39-8.88)	*3.98(2.28)* *3.59(1.47-8.10)*
Testosterone, nmol/L	21.02(7.56) **^a^** 19.88(10.80-34.54)	20.09(7.13) **^a^** 19.24(10.12-33.24)	20.66(7.85) **^a^** 19.55(10.58-37.48)	*20.76(7.51)* *19.68(10.60-34.33)*
Estradiol, nmol/L	0.204(0.064) **^ab^** 0.195(0.113-0.317)	0.213(0.059) **^b^** 0.207(0.119-0.330)	0.194(0.049) **^a^** 0.192(0.122-0.284)	*0.204(0.061)* *0.197(0.115-0.314)*
Inhibin B, pg/mL	184.5(63.8) **^a^** 174.2(98.3-300.3)	172.7(58.8) **^b^** 168.7(84.4-274.8)	171.0(72.5) **^b^** 164.0(61.1-311.5)	*178.4(65.0)* *171.5(83.3-295.6)*
TG, mmol/L	1.06(0.65) **^a^** 0.87(0.39-2.34)	1.12(0.73) **^a^** 0.93(0.40-2.73)	1.30(0.78) **^b^** 1.06(0.43-2.90)	*1.10(0.68)* *0.91(0.39-2.39)*
TC, mmol/L	3.88(0.89) **^a^** 3.77(2.65-5.46)	3.93(0.90) **^a^** 3.84(2.75-5.57)	4.00(0.86) **^a^** 3.89(2.77-5.52)	*3.90(0.88)* *3.80(2.67-5.47)*
HDL-C, mmol/L	1.16(0.34) **^a^** 1.12(0.70-1.74)	1.14(0.32) **^a^** 1.11(0.70-1.70)	1.08(0.31) **^b^** 1.03(0.65-1.56)	*1.15(0.33)* *1.11(0.69-1.73)*
LDL-C, mmol/L	2.24(0.89) **^a^** 2.11(1.03-3.82)	2.27(0.85) **^a^** 2.22(1.10-3.90)	2.33(0.81) **^a^** 2.27(1.19-3.72)	*2.26(0.86)* *2.15(1.07-3.82)*
Fasting glucose, mmol/L	4.8(0.9) **^a^** 4.7(3.6-6.1)	4.9(1.0) **^a^** 4.8(3.6-6.5)	5.0(1.0) **^a^** 4.8(3.6-6.8)	*4.8(0.9)* *4.8(3.6-6.3)*
Uric acid, µmol/L	350(92) **^a^** 340(225-516)	347(90) **^a^** 333(225-498)	365(101) **^b^** 354(246-540)	*352(93)* *340(226-518)*
Serum zinc concentration, μmol/L	22.5(9.0) **^a^** 20.2(13.7-39.2)	22.0(8.5) **^a^** 19.7(13.4-40.9)	25.2(11.8) **^b^** 21.2(14.1-51.3)	*22.8(9.4)* *20.1(13.7-41.3)*
Seminal zinc concentration, μmol/L	1589(1001) **^a^** 1423(395-3527)	1474(923) **^ab^** 1233(354-3469)	1392(954) **^b^** 1133(336-3042)	*1536(980)* *1325(372-3487)*
Seminal zinc content, μmol/ejaculate	5.77(4.65) **^a^** 4.45(1.07-14.04)	5.03(4.11) **^ab^** 3.85(0.78-11.99)	4.52(3.53) **^b^** 3.68(0.85-11.20)	*5.43(4.41)* *4.20(0.94-13.19)*

Results based on raw data. Values are presented as mean (SD) and median (5-95th percentile). BMI, body mass index; BTV, paired testicular volume; TZI, teratozoospermia index; DFI, sperm DNA fragmentation index; LH, luteinizing hormone; FSH, follicle-stimulating hormone; TG, triglycerides; TC, total cholesterol; HDL-C, high-density lipoprotein cholesterol; LDL-C, low-density lipoprotein cholesterol; a, b - comparisons with different superscripts within variable are significant (p<0.05).

### Comparison of anthropometric, semen, hormonal, and metabolic parameters between groups of participants with different cigarette smoking status

Using our group of non-smokers as a reference group, we found no statistically significant differences between non-smokers and both groups of smoker in height, BTV, serum levels of LH, FSH, testosterone, total cholesterol, LDL-C, fasting glucose (**Table 1**). The medians of age, body weight, waist and hip circumference, BMI in heavy smokers were significantly higher than in non-smokers (p<0.05, **Table 1**). The smoking intensity (the number of cigarettes smoked per day) was 2.7 times higher in heavy smokers than in moderate smokers (p<0.05, **Table 1**).

Current smokers of both consumption categories had reduced semen volume, total sperm count, sperm concentration and the inhibin B level compared to non-smokers, while only heavy smokers had lower sperm progressive motility and higher TZI and DFI% compared to non-smokers (p<0.05, **Table 1**). In general, the heavy smokers had worse semen quality than moderate smokers or nonsmokers. The group of heavy smokers was also characterized by an impaired metabolic profile: the serum TG and uric acid levels were significantly higher, and the HDL-C level was significantly lower compared with the group of non-smokers (p<0.05, **Table 1**). In addition, the serum zinc concentration was significantly higher, while the seminal zinc concentration and content were significantly lower in heavy smokers compared with non-smokers (p<0.05, **Table 1**). On the contrary, the group of moderate smokers was very close to non-smokers in many respects, with the exception of most semen parameters and the inhibin B level (**Table 1**).

### Comparison of smokers and non-smokers by anthropometric, seminal, hormonal, zinc and metabolic parameters in different ethnic groups

Three of the most numerous ethnic groups were selected from the entire studied population: Slavs, Buryats and Yakuts. The ethnic differences in anthropometric, semen and reproductive hormone parameters in men of these three groups have been described elsewhere (73). Briefly, the higher semen quality was found in Slavs, the average in Buryats and the lowest in Yakuts due to the higher testicular function in Slavs compared to Asians. Some anthropometric indicators (weight, height, waist and hip circumference) were higher for Slavs than for Buryats, and weight and height were higher for Slavs than for Yakuts. In addition to published ethnic data, we included in this study the metabolic and zinc parameters, and smoking consumption. The proportion of male smokers and the smoking intensity (the number of cigarettes smoked per day) differed significantly between ethnic groups (p<0.05, **Table 2**). The prevalence of cigarette smoking was 32.1% among Slavs, 46.6% among Buryats, 44.8% among Yakuts, the latter two did not differ from each other. The median smoking intensity was the highest among the Slavs, moderate among the Buryats and Yakuts, the latter two did not differ from each other (12.5; 9.0; 6.3 cigarettes per day, respectively).

**Table 2:** Comparison of smokers and non-smokers by anthropometric, seminal, hormonal, zinc and metabolic parameters in different ethnic groups.

Parameters	Buryats (n=191)		Slavs (n=654)		Yakuts (n=125)	
	Non-smokers (n=102)	Smokers (n=89; 46.6%)	Non-smokers (n=444)	Smokers (n=210; 32.1%)	Non-smokers (n=69)	Smokers (n=56; 44.8%)
Age, years	23.6(6.6) 21.0(18.0-35.0)	24.4(6.6) 22.0(18.0-38.0)	25.6(7.5) 23.0 (18.0-40.0)	26.1(7.7) 24.0 (19.0-40.0)	24.5(6.4) 22.0 (18.0-33.0)	26.2(7.0) 26.0 (18.0-39.0)
Body weight, kg	70.0(11.7) 67.0(56.0-88.0)	71.6(15.1) 70.0 (53.0-95.5)	78.1(13.7) 75.0 (60.0-103.2)	78.7(14.3) 77.0(60.0-105.0)	68.4(12.5) 65.0 (50.0-90.0)	74.9(14.7) * 73.0 (55.0-106.0)
Height, cm	175.1(6.6) 174.0(165.0-187.0)	174.4(6.1) 174.0 (165.0-184.0)	179.4(6.9) 180.0 (169.0-190.0)	178.5(6.6) (168.0-190.0)	171.8(172.0) 162.0(181.0-5.6)	173.0(6.7) 173.0 (161.0-182.0)
Waist circumference, cm	81.2(10.7) 78.5(68.0-101.0)	83.1(13.9) 82.0 (67.0-103.0)	84.3(10.2) 82.0 (71.0-104.0)	86.2(11.1) * 85.0 (71.0-105.0)	83.0(11.0) 81.0 (68.0-103.0)	88.5(13.7) * 86.5 (69.0-112.0)
Hip circumference, cm	93.6(6.6) 92.0(84.0-104.0)	94.6(8.3) (94.084.0-106.0)	99.2(8.0) 98.0 (88.0-113.0)	99.3(8.4) 99.5 (87.0-112.0)	96.5(7.0) 96.0 (87.0-108.0)	98.8(8.6) 96.0 (88.0-116.0)
BMI, kg/m^2^	22.8(3.7) 21.8(18.4-30.8)	23.6(5.0) 23.0 (17.2-30.6)	24.2(3.8) 23.7 (19.2-31.4)	24.7(4.1) 24.2 (18.9-31.2)	23.2(4.0) 22.9 (17.4-31.1)	25.1(4.9) * 23.8 (18.4-35.3)
BTV, ml	36.2(6.9) 38.3(25.0-50.0)	37.0(5.8) 40.0 (27.0-45.0)	40.9(8.6) 40.0 (27.0-55.0)	41.8(8.8) 40.0 (30.0-60.0)	36.9(9.1) 36.0 (29.0-56.0)	36.6(6.7) 36.0 (28.0-48.0)
Smoking, sigarettes/day	0	9.1(5.4) 9.0 (2.0-20.0)	0	12.7(6.8) 12.5 (2.0-20.0)	0	8.2(5.6) 6.3 (1.5-20.0)
Semen volume, ml	3.2(1.2) 3.0(1.5-5.3)	3.1(1.4) 2.8 (1.3-5.9)	3.8(1.7) 3.7 (1.5-6.6)	3.4(1.8) * 3.0 (1.1-6.6)	3.0(1.1) 3.0 (1.5-5.3)	3.1(1.4) 2.9 (1.1-5.9)
Total sperm count, ×10^6/^ejaculate	159.7(140.0) 124.9(33.6-379.9)	129.7(106.3) 103.9 (14.8-370.3)	217.2(202.2) 178.2 (27.4-462.6)	167.8(139.7) * 136.5 (5.8-455.6)	104.2(85.5) 84.8 (16.6-244.2)	106.1(80.7) 79.6 (8.4-272.4)
Sperm concentration, ×10^6^/mL	50.89(38.45) 41.16(14.00-120.00)	42.78(30.87) 38.00 (6.37-97.38)	59.99(43.95) 49.09 (10.38-149.18)	52.40(39.70) * 43.38 (3.94-134.52)	36.34(26.04) 33.13 (5.50-92.13)	35.79(26.78) 30.80 (4.50-96.14)
Progressive motility, %	49.8(25.7) 46.1(10.3-89.9)	46.5(27.9) 46.5 (2.8-88.1)	46.7(26.3) 44.3 (5.8-90.6)	41.0(27.2) * 37.8 (2.9-87.3)	37.2(21.0) 34.0 (8.7-73.2)	38.2(24.5) 37.9 (5.8-90.1)
Normal morphology, %	7.0(2.7) 7.0(2.9-11.9)	6.8(2.6) 6.5 (2.8-11.3)	7.2(3.1) 7.0 (2.6-12.8)	6.8(3.3) 6.8 (1.9-11.8)	4.6(2.3) 4.8 (1.0-8.5)	5.3(2.4) 5.3 (1.3-9.5)
TZI	1.49(0.12) 1.47(1.30-1.66)	1.47(0.10) 1.45 (1.33-1.64)	1.48(0.11) 1.47 (1.32-1.69)	1.50(0.13) * 1.49 (1.31-1.76)	1.52(0.12) 1.52 (1.35-1.73)	1.53(0.11) 1.51 (1.33-1.74)
DFI, %	**-**	**-**	8.29(5.53) (n=138) 6.81(2.70-20.99)	13.15 (11.24) * (n=49) 8.63 (2.69-36.89)	**-**	**-**
LH, mIU/mL	3.81(1.41) 3.43(2.02-6.05)	4.06(1.59) 3.75 (1.92-6.82)	3.45(1.38) 3.20 (1.61-6.09)	3.60(1.47) 3.33 (1.48-6.40)	3.42(1.40) 3.09 (1.53-6.19)	3.84(1.72) 3.95 (1.25-7.02)
FSH, mIU/mL	4.58(2.28) 4.17 (1.86-8.32)	4.24(1.99) 3.95 (1.56-8.80)	3.69(2.31) 3.32 (1.45-7.86)	3.55(1.87) 3.16 (1.29-6.91)	4.67(1.70) 4.53 (2.00-7.53)	5.50(3.70) 4.73 (1.91-12.06)
Testosterone, nmol/L	19.05(6.19) 17.74 (11.37-31.31)	18.24(5.39) 17.67 (9.71-27.98)	21.21(7.51) 20.29 (10.54-34.54)	21.46(8.28) 20.44 (10.55-37.12)	21.32(7.60) 21.87 (10.80-33.36)	18.83(6.52) * 17.57 (10.52-31.79)
Estradiol, nmol/L	0.235(0.068) 0.220 (0.163-0.351)	0.222(0.048) 0.204 (0.162-0.298)	0.192(0.061) 0.182(0.107-0.312)	0.192(0.058) 0.186 (0.114-0.297)	0.234(0.062) 0.217 (0.162-0.354)	0.220(0.044) 0.212 (0.160-0.293)
Inhibin B, pg/mL	144.7(56.1) 146.2 (43.8-245.6)	155.5(64.0) 153.9 (51.5-258.8)	193.5(64.8) 184.3 (104.2-305.7)	183.5(68.1) 179.9 (88.7-307.3)	172.6(46.3) 163.1 (111.1-271.7)	149.4(49.0) * 156.3 (66.1-227.6)
TG, mmol/L	1.04(0.71) 0.84 (0.38-2.35)	1.04(0.70) 0.85 (0.44-2.25)	1.09(0.68) 0.87 (0.39-2.49)	1.27(0.86) * 1.02 (0.41-3.12)	1.04(0.59) 0.85 (0.44-2.33)	1.30(0.66) * 1.16 (0.43-2.90)
TC, mmol/L	3.73(0.81) 3.56 (2.60-5.13)	3.85(0.85) 3.82 (2.65-5.38)	3.87(0.88) 3.79 (2.66-5.48)	3.99(0.91) 3.93 (2.75-5.74)	4.01(1.02) 3.95 (2.67-6.80)	4.04(0.81) 4.02 (2.94-5.35)
HDL-C, mmol/L	1.04(0.26) 1.03 (0.69-1.54)	1.08(0.29) 1.04 (0.70-1.66)	1.18(0.36) 1.14 (0.69-1.75)	1.15(0.32) 1.12 (0.66-1.69)	1.16(0.33) 1.11 (0.68-1.82)	1.12(0.35) 1.08 (0.68-1.69)
LDL-C, mmol/L	2.21(0.72) 2.07 (1.25-3.57)	2.29(0.81) 2.26 (1.13-3.86)	2.20(0.89) 2.12 (0.99-3.77)	2.27(0.85) 2.22 (1.08-3.76)	2.38(1.04) 2.15 (1.15-4.91)	2.34(0.76) 2.29 (1.23-3.76)
Fasting glucose, mmol/L	4.9(0.9) 4.8 (3.7-6.6)	4.9(6.4) 0.9 (4.8 -3.5)	4.8(1.0) 4.7 (3.6-6.2)	5.0(0.9) * 4.8 (3.7-6.8)	4.6(0.7) 4.5 (3.3-5.7)	4.9(1.4) 4.9 (3.3-8.0)
Uric acid, μmol/L	346(73) 340(229-470)	344(78) 333 (238-495)	355(91) 345 (225-526)	369(102) 355 (240-539)	333(112) 319 (197-476)	345(105) 332 (208-512)
Serum zinc concentration, μmol/L	19.6(7.5) 17.4(12.9-31.5)	21.3(10.8) 17.5 (12.8-49.5)	23.9(9.9) 20.9 (14.8-42.5)	25.6(11.2) 21.6 (14.9-50.0)	20.5(6.9) 18.6 (12.5-35.3)	21.3(7.1) 19.2 (14.0-32.2)
Seminal zinc concentration, μmol/L	1419(869) 1181(355-2999)	1522(968) 1159 (354-3925)	1725(1077) 1514 (398-3882)	1443(995) * 1162 (348-3483)	1368(979) 1128 (395-2643)	1531(752) 1440 (460-3139)
Seminal zinc content, μmol/ejaculate	4.41(3.20) 3.69 (0.68-11.22)	4.60(3.89) 3.42 (0.89-11.99)	6.46(5.12) 5.16(1.30-15.49)	5.11(4.39) * 3.76 (0.85-12.03)	4.13(2.96) 3.23(0.81-9.57)	4.94(3.06) 4.19(0.90-10.74)

Results based on raw data. Values are presented as mean (SD) and median (5-95th percentile); BMI, body mass index; BTV, paired testicular volume. TZI, teratozoospermia index; DFI, DNA fragmentation index; LH, luteinizing hormone; FSH, follicle-stimulating hormone; TG, triglycerides; TC, total cholesterol; HDL-C, high-density lipoprotein cholesterol; LDL-C, low-density lipoprotein cholesterol; * - differences between groups of smokers and non-smokers within each ethnic group are significant (p<0.05).

The effect of cigarette smoking on anthropometric, seminal, hormonal, zinc and metabolic parameters in men of different ethnic groups is presented in **Table 2**. Two categories of smokers were combined in each ethnic group due to the small number of heavy smokers among the Buryats and Yakuts. In the Buryat group, smokers did not differ from non-smokers in all the parameters studied (**Table 2**). In the Yakut group, smokers had higher weight, waist circumference, BMI and TG level, as well as lower testosterone and inhibin B levels compared with non-smokers (p<0.05, **Table 2**). In the Slavic group, smokers had significantly reduced semen volume, total sperm count, sperm concentration, progressive motility, and significantly increased TZI and DFI% compared with non-smokers (p<0.05, **Table 2**). In smoking Slavic men, the impairment in semen quality was associated with a decrease in the seminal zinc concentration and content (p<0.05, **Table 2**). In addition, in this ethnic group there was a significant increase in waist circumference, the triglyceride and glucose levels in smokers compared to non-smokers indicating a metabolic disorder (p<0.05, **Table 2**). Thus, the most pronounced negative effects of cigarette smoking were found in Slavs, weaker negative effects were found in Yakuts, and no negative effects of smoking were found in Buryats.

## Discussion

Our study is a rather large-scale observational investigation of the effects of tobacco smoking on semen quality, hormonal, zinc and metabolic parameters in men from Russia. According to our data, the prevalence of smoking among Russian men with a median age of 23 years was 36.8% and coincided with WHO data (14) for the world population (36.7%). In the first part of our study, we studied the effect of cigarette smoking in the multi-ethnic study population, whereas in the second part we focused on the reproductive consequences of smoking in selected ethnic groups. The first part of the study included two categories of cigarette smoking (moderate smokers and heavy smokers) to examine the dose-dependent associations between cigarette smoking and the indicators studied. The most important consequences of cigarette smoking were lower semen parameters, including semen volume, total sperm count, sperm concentration and progressive motility, increased sperm DNA fragmentation and teratozoospermia. Our results on men from the general population are consistent with conclusions of many other studies in infertile men (21, 33–36, 81–83), in men from the general population (32, 51, 54, 84, 85) or in case-control studies (29–31, 38, 39). Although the mechanisms by which smoking may be linked to detrimental effects on semen parameters remain to be fully elucidated, they are due to a direct exposure of the toxic content of cigarette smoke (mutagens, carcinogens, nicotine and its metabolites, heavy metals) (12, 20, 22, 81). An additional way may be associated with the direct effect of tobacco components, including nicotine, on the function of accessory glands (seminal vesicles and prostate gland), which alter the semen volume and the functional properties of sperm (12, 19, 20, 81, 86). Moreover, oxygen deficiency produced by cigarette smoking affects spermatogenesis, resulting in impaired sperm production (9, 81).

In our study, we also investigated how smoking affects sperm DNA fragmentation in men. It was shown that DFI% increased by about 75% in heavy smokers compared to non-smokers. These findings are consistent with the results of previous reports (43, 64, 87, 88) and confirm the conclusions of recent reviews (12, 20, 22, 41, 42, 81). Most studies have shown that the negative effect of smoking on sperm DNA can mainly be caused by excessive production of reactive oxygen species (ROS) (20, 22, 39, 43, 81), assuming that the increase in sperm DNA fragmentation in heavy smokers in our study may also be caused by an imbalance between antioxidant and pro-oxidant factors.

In our multi-ethnic study population, tobacco smoking did not cause an imbalance in the LH, FSH, testosterone and estradiol levels involved in the control of spermatogenesis, with the exception of inhibin B. These results are consistent with previously reported (21, 54, 55, 61). The hormonal results of our study are in line with the results of two population-based studies, which did not reveal a significant effect of smoking on the levels of testosterone, estradiol, inhibin B, sex hormone binding globulin, LH and/or FSH in young men (54, 55). However, as a rule, studies show contradictory associations between smoking and reproductive hormones (48, 49), and both decreased (38, 53, 63) and increased (50–52, 56, 58, 62) testosterone, LH and/or FSH levels have been described in smokers as compared to non-smokers.

The results of our study showed that cigarette smoking had an adverse effect on the metabolic health of smokers (an increase in triglycerides and uric acid, as well as a decrease in the HDL-C), which corresponds to the data obtained in other studies (71, 89–91). A meta-analysis conducted by Kauss and co-authors demonstrated that smoking was also associated with a decrease in the levels of apolipoproteins AI and AII and an increase in the level of apolipoprotein B, thereby confirming the negative impact of smoking on lipid metabolism (92). A possible mechanism for how cigarette smoking can alter the lipid profile has been proposed (92, 93). During cigarette smoking, nicotine stimulates the secretion of catecholamines and cortisol, activating adenylcyclase in adipose tissue, which leads to both lipolysis and the release of free fatty acids into the circulation, as well as to an increase in the lipoprotein production, including the HDL concentration. But, on the other hand, the release of catecholamines leads an increase in heart rate, blood pressure and cardiac output and increases the risk of cardiovascular disease. In our study, the combination of an altered lipid profile and increased body weight, waist circumference and BMI, which we found in heavy smokers, may further increase the risk of cardiovascular diseases.

It is considered proven that many of the harmful consequences of cigarette smoking are due to smoking-induced increase in the production of reactive oxygen species, leading to oxidative stress (9, 13, 20, 22, 39, 43, 64, 94, 95). The data obtained in our study correspond to the idea that smoking-induced oxidative stress appears to be the main mediating mechanism of the damaging effects of tobacco on male reproductive function, leading to the reduced semen quality, sperm DNA fragmentation, as well as an imbalance of lipid profile, which ultimately weakens male fertility.

Despite a fairly large amount of data on the impact of cigarette smoking on men’s reproductive health, often authors receive conflicting data, the interpretation of which is ambiguous and does not reach consensus. Probably, the magnitude of the negative effect depends on environmental and climatic conditions, ethnic origin and cultural traditions of the population, and, most likely, the effect will manifest itself with pronounced and prolonged exposure. There are also some uncertainties regarding the methodical questions, in particular, the dose-dependent effects of smoking on male reproductive health, which may partially explain the inconsistency of the semen and hormonal results. For example, some researchers classify smokers as those who smoke from 1 to 15 cigarettes per day, while others take into account smoking experience and include in the group of male smokers only those who regularly smoke during the year (23).

In the second part of our study, we tried to find out how smoking affects reproduction-related parameters in various ethnic groups. Men of three ethnic groups - Buryats, Slavs and Yakuts - were selected from our multi-ethnic study population. Our previous studies have demonstrated significant reproductive differences between these ethnic groups on semen quality, hormonal levels, and genetic factors (73, 74). In the present study, we have shown that the proportion of smokers was the highest in the Buryat group and the lowest among the Slavs, the Yakuts occupied an intermediate position, but the smoking intensity (cigarettes per day) was the highest among the Slavs and moderate among the Buryats and Yakuts, who did not differ in this parameter. The data obtained suggest a greater tobacco addiction in Slavs than in both Asian groups. Moreover, the most pronounced negative effects of cigarette smoking were found in men of Slavic ethnicity, weaker in Yakuts, and no negative effects were found in Buryats. The main harmful impact of smoking on reproductive function was noted among Slavs, including semen quality, sperm DNA fragmentation, seminal zinc content and metabolic profile.

In our previous study, we found that semen parameters were close and positively related to seminal zinc content, while seminal zinc deficiency was associated with a decrease in semen quality (68). In this study, we showed that in the entire study population and in a separate Slavic group, smokers had both a lower seminal zinc content and impaired semen parameters, including increased sperm DNA fragmentation, compared with non-smokers. Previously published studies have also indicated the adverse impact of smoking on the seminal zinc level (64–67, 69, 70). Our current zinc data coupled with previous published data suggest that seminal zinc is involved in the effect of cigarette smoking on semen parameters. Zinc is a cofactor for various proteins and part of the antioxidant defense, playing an important role in the inactivation of excess reactive oxygen species (ROS) in seminal plasma, as reported by recent reviews (96, 97). It seems obvious that cigarette smoking reduces the seminal zinc level due to increased ROS production and leads to depletion of the semen antioxidant defense.

The ethno-specific effects of cigarette smoking identified in this study may be associated with higher cigarette consumption and, consequently, with more harmful effect of nicotine and other tobacco compounds in the Slavic group compared to both Asian groups, in which smoking did not lead to severe damage. Moreover, taking into account the smoking-induced oxidative stress as the main damaging mechanism, it can be assumed that the ethno-specific effects of smoking may be due to a different genetic background and are connected with genetic differences in the activity of antioxidant enzymes. The seminal plasma possesses an antioxidant system capable of counteracting the harmful effects of ROS, but smoking alters the balance between total antioxidant capacity and ROS production, increasing lipid peroxidation and reducing antioxidant activity, including seminal zinc, vitamin C, and enzymes (superoxide dismutase containing zinc, glutathione peroxidase, catalase) and others antioxidants (20, 95–97). In our previous study, we compared the level of lipid peroxidation and antioxidants in the seminal plasma of young Buryats and Slavs (98). The results showed a more active functioning of the antioxidant system in the Buryats compared to the Slavs. In the present study, a decrease in the seminal zinc level of smoking Slavs compared to non-smokers is an additional evidence of a decline in semen antioxidant defense. Our study highlighted the role of the genetic background in the reproductive effects of smoking, presumably genes involved in antioxidant defense. One study showed that the combination of smoking and one functional polymorphism of the antioxidant gene NRF2 (erythroid 2-related factor 2) led to a significant decrease in semen quality, a fact that is in line with our above assumption (99).

Further research is needed to elucidate the ethno-specific molecular pathways by which cigarette smoking can affect spermatogenesis, which could also explain the conflicting data on the detrimental effects of cigarette smoking on semen quality. Returning to the underlying lifestyle causes of adverse temporal trends in the male reproductive potential, which were first reported more than half a century ago, we can assume, based on the facts obtained in this study, a significant contribution of tobacco smoking to this phenomenon, taking into account its ethno specific features.

A strength of the study is the rather large cohort of men with detailed demographic characteristics, ethnic origin, lifestyle factors, including smoking, past and current diseases for each participant. In the study, we evaluated a wide range of 30 anthropometric, seminal, hormonal, zinc and metabolic parameters in order to more comprehensively analyze the reproductive consequences of cigarette smoking. In addition, we included in the study young men from the general population, who were quite similar in social status; used a standardized recruitment protocol and questionnaire; all samples were collected and processed by the same scientific team using the same laboratory methods, equipment and supplies. The large study population allowed us to adjust the data for known confounders when comparing groups. Given the wide diversity of nationalities in Russia, we took this opportunity to clarify the ethno-specific impact of cigarette smoking on reproduction-related indicators, which can deepen existing knowledge about the reproductive consequences of smoking and have obvious advantages when integrating the results into clinical practice and research.

The study has some limitations. Our study is retrospective and observational in nature, which did not allow us to draw exhaustive conclusions about mediating mechanisms. In addition, cigarette smoking status was self-reported, resulting in risk of bias. It was difficult to accurately determine the time frame for starting smoking, since the participants, as a rule, gradually and intermittently became involved in tobacco addiction. Furthermore, this study did not analyze antioxidants and antioxidant enzymes other than zinc to determine the extent of damage of the antioxidant system, especially in heavy smokers. Finally, the number of Buryat and Yakut smokers, as well as their data on sperm DNA fragmentation, have been limited and should be expanded in future studies.

## Conclusion

Our study showed the negative impact of cigarette smoking habits on male reproductive potential in adult Russian men from the general multi-ethnic population. In smokers, who smoked more than 10 cigarettes a day, we observed reduced semen volume, total sperm count, sperm concentration, progressive motility, seminal zinc levels, increased sperm DNA fragmentation and teratozoospermia, as well as metabolic imbalance. When investigating the effects of smoking in the selected ethnic groups, the most pronounced negative effects were found in Slavs, weaker effects in Yakuts, and no effects in Buryats. Reduced semen quality and seminal zinc content, increased sperm DNA fragmentation and teratozoospermia, serum levels of triglycerides and fasting glucose were detected in Slavic smokers, while Yakut smokers demonstrated only a slight decrease in testosterone and inhibin B levels as well as an increase in triglycerides. The data obtained indicate the ethno-specific effect of cigarette smoking on the male reproductive potential and suggest that ethnic differences may be due to a genetic background, presumably genes regulating the antioxidant system, taking into account smoking-induced oxidative stress as the main mediating mechanism.

## Data availability statement

The raw data supporting the conclusions of this article will be made available by the authors, without undue reservation.

## Ethics statement

The studies involving humans were approved by Research Institute of Clinical and Experimental Lymрhology – Branch of the Institute of Cytology and Genetics, Siberian Branch of Russian Academy of Sciences (RICEL- Branch of IC&G SB RAS). The studies were conducted in accordance with the local legislation and institutional requirements. The participants provided their written informed consent to participate in this study.

## Author contributions

LO: Conceptualization, Funding acquisition, Methodology, Supervision, Writing – original draft, Writing – review & editing. MK: Formal Analysis, Investigation, Writing – original draft. AO: Conceptualization, Data curation, Methodology, Resources, Software, Writing – original draft.
